# Comprehensive comparison between conservative therapy and surgical management for completely displaced and comminuted mid-shaft clavicle fractures

**DOI:** 10.1007/s00264-024-06198-1

**Published:** 2024-05-07

**Authors:** Xiao Han, Yufu Zhang, Xigong Zhang, Jie Tan

**Affiliations:** 1 Department of Orthopedic Trauma, Beijing Jishuitan Hospital, Capital Medical University, Xicheng District, Beijing, China; 2 Department of Orthopedic Surgery, Beijing Jishuitan Hospital, Peking University Fourth School of Clinical Medicine, Xicheng District, Beijing, China

**Keywords:** Mid-shaft clavicle fracture, Operative, Non0operative, Union, Outcome

## Abstract

**Purpose:**

It is still controversial whether complete displaced mid-shaft clavicle fractures should be treated with internal fixation or conservative therapy. This retrospective study aims to compare clinical outcomes of two treatment protocols.

**Materials and methods:**

105 patients with displaced and comminuted mid-shaft clavicle fractures were included in this study, among which 55 patients were treated conservatively and 50 patients accepted surgical fixation and were followed up for over 20 months on average. Rate of union, malunion, time taken for union, functional outcome, self-reported satisfaction and complications were compared.

**Results:**

Union rate of operative group (*n*=49, 98.0%) was higher than the non-operative group (*n*=48, 87.3%). Time taken for union in operative group (2.37±1.06 months) was shorter than the non-operative group (3.69±1.01 months). Malunion and asymmetric were only seen in the conservative group. Numbness of the shoulder was only reported in the operative group (*n*=23, 46.0%). Most of patients in the operative group (*n*=45, 90%) accepted a second operation to remove the implant. No statistically difference was found in self-reported satisfaction, Quick-DASH and Constant-Murley score. The operative group returned to work faster (1.47±0.89 to 3.34±1.37 months), regained full range of motion earlier (1.66±0.78 to 3.83±1.24 months) and regained strength faster (3.86±2.45 to 8.03±2.78 months) than the non-operative group.

**Conclusion:**

Complete displaced and comminuted mid-shaft clavicle fractures treated surgically have more reliable union and faster recovery when compared to conservatively treated fractures.

**Supplementary Information:**

The online version contains supplementary material available at 10.1007/s00264-024-06198-1.

## Purpose

Clavicle fractures account for 2.6-4% of all fractures, with over 80% of these injuries occurring at the middle shaft [1]. In recent years, the popularity of extreme sports has led to an increase in clavicle fractures, which are typically caused by deceleration injuries to the shoulder. Emergency visits have seen a higher occurrence of these fractures, with 6.2% of clinic visits at ski resorts being attributed to clavicle fractures [2]. However, the management of mid-shaft clavicle fractures remains a topic of controversy [3]. In conventional opinion, midshaft clavicle fractures without intensive indications for surgical intervention, such as open or potential open fractures, neurovascular deficits, or multiple traumas, are typically treated with conservative therapy. However, lower healing incidence, longer rehabilitation time, and intolerable suffering have sometimes led patients to seek operative solutions [4].

Several factors should be taken into consideration when making clinical decisions, including union, complications, cost (both financial and time-related), functionality, and cosmetic concerns. In our efficiency-oriented modern society, there is increasing attention on faster return to work, sports, and social contact, which has made operative treatment more popular for clavicle fractures [5]. The superiority of surgical therapy over non-surgical therapy for midshaft clavicle fractures, particularly in the middle and long term, is still a topic of controversy. Previous studies have provided inconclusive suggestions due to differences in candidates, surgical techniques, patient compliance, follow-up time, observation targets, and statistical methods, which have led to varying and even contradictory conclusions [6, 7]. Furthermore, although the cosmetic issue of clavicle fractures has been noted before, there has never been a direct comparison between conservative and operative patients [8].

In this retrospective study conducted at a single centre, our objective was to examine the disparity in bone healing, clinical function, self-reported satisfaction, and complications between the conservative and operative groups. We suppose that both therapies have its advantages and disadvantages, which should be considered comprehensively. The findings of this study would significantly contribute to providing robust evidence for making clinical decisions.

## Materials and methods

Patients diagnosed with complete displaced midshaft clavicle fractures (Robinson 2B type) at the Emergency of Beijing Jishuitan Hospital from August 1 2021 to December 31 2022 were enrolled in this retrospective research.

The inclusion criteria were as follows: 1) diagnosis of a middle shaft fracture with complete separation of two segments (Robinson type 2B), with a displacement distance of more than 1.5cm; 2) participants aged above 18 and below 60 years; 3) interval from primary injury to diagnosis of less than three days; 4) no other interventions from the time of injury to diagnosis; 5) participants who fully understood and strictly followed the conservative treatment plan or agreed to undergo surgery at our medical centre; 6) average follow-up period of 21.24(8-33) months; 7) participants who fully understood the content of the study and provided informed consent; 8) participants who were able to complete all questionnaires and provide necessary materials for follow-up.

The exclusion criteria were: 1) any definite indication for surgery as mentioned above; 2) pathological fracture; 3) presence of craniocerebral injury, burns, or any other injury requiring clinical intervention or that might influence the functional and/or cosmetic evaluation; 4) any non-compliance with the inclusion criteria.

All patients were fully informed about the advantages and disadvantages of both conservative and surgical management based on conclusions reported in previous literatures. The final treatment choice was made by the patients. Based on their treatment decision, the patients were divided into non-operative and operative groups. The study was an observational and retrospective study without any randomization.

Ethics approval was obtained from the ethics committee of Beijing Jishuitan Hospital and written informed consent for participation and publication was obtained from the patient. All methods were performed in accordance with the relevant guidelines and regulations of Beijing Jishuitan Hospital.

### Treatment protocol

The surgical procedure involved the following steps: 1) The skin and subcutaneous tissues were incised layer by layer using a supraclavicular approach. 2) The end of the fracture was exposed while ensuring the protection of the cutaneous nerve. 3) The clavicle shaft and free bone fragments were reduced. 4) Fixation was achieved with a variable-angle locking compression plate (DePuy Synthes, Warsaw, IN, USA); 5) Intraoperative fluoroscopy was performed to confirm satisfactory reduction, and passive movement of the shoulder was conducted to check stability. 7) The area was rinsed, sutured, and dressed. All operations were performed by a single medical team comprising one professor of traumatic orthopaedics and one resident.

The conservative procedure consisted of 1) Application of a “figure-of-eight” brace and sling. 2) Radiological examination to confirm acceptable reduction. 3) Step analgesia regimen including NSAIDS, weak opioid analgesics, and strong opioid analgesics. 4) Avoidance of weightlifting and continuous use of the brace for at least four weeks.

### Follow-up and rehabilitation

Regular follow-up of both groups was conducted at our centre and any complications were recorded, along with the subsequent management. The average follow-up period in operative group was 20.34 months (ranging from 20 to 24months), and figures for non-operative group was 22.05 months (ranging from 8 to 33months). In the operative group, early active activity was encouraged, but the range of flexion and abduction was limited to 90 degrees within four weeks post-operation. Full range of movement was advised six weeks after the operation, and sports activities were typically permitted after three months post-operation. Rehabilitation exercises for the non-operative group began after callus formation, and resistance movements commenced after clinical union.

The radiological assessment was conducted at every follow-up in outpatient clinic by specialized surgeon, and x-ray was taken to check the implant position, malunion, formation of callus while assessing nonunion (those not achieved union even at 9 months). The function was assessed at the final follow-up with the help of Quick DASH and Constant-Murley questionnaire. Recovery of work, strength and full range of movement (ROM) as well as time cost of above were recorded Patient satisfaction was also assessed in terms of overall satisfaction, cosmetic satisfaction and pain level, and those were all achieved by visual analogue scale. Complications of conservative group were malunion, ununion and neurological symptoms caused by pressure of the bump. The complications of the operative patients included infection, failure of implant, undesirable healing, decrease of sensation of clavicle region, nonunion and malunion.

### Statistical methods

Above all, Shapiro–Wilk normality test was performed to judge the normality of continuous quantitative data, including age, follow-up time, time needed for union, VAS of pain, VAS of self-satisfaction, VAS of cosmetic degree, Constant-Murley score, Quick-DASH score, time needed for return to work, and time needed for regain of ROM. Categorical variables were tested with two independent samples chi-square test. Normally distributed continuous variables were tested with t-test for two independent samples. Quantitative data that were not normally distributed was tested by nonparametric Wilcoxon rank-sum test for two independent samples. Statistical analysis was performed using SPSS software (version 20). The difference was considered statistically significant when *P* < 0.05.

## Results

### General information

The study included a total of 105 patients, consisting of 83 males and 22 females. Among them, 55 cases were in the non-operative group and 50 cases were in the operative group. There were no significant differences found regarding the demographics of these patients (Table 1). In non-operative group, the average age was 41 years and 16.4% of patients were female, and figures for operative group were 39 years and 26.0% respectively. The follow-up time did not exhibit any noticeable difference, with 22.05±1.18 months for the non-operative group and 20.34±7.28 months for the operative group (*p*=0.791).

**Table 1:** Comparison of operative group and non-operative group

	Non-operative group	Operative group	*P*
	(*n*=55)	(*n*=50)	
Gender (female)	9 (16.4%)	13 (26.0%)	0.166
Age (years)	40.62±10.44	39.28±11.17	0.527
Side (left)	37 (67.3%)	27 (54.0%)	0.117
Follow-up time (month)	22.05±1.18	20.34±7.28	0.791
Union	48 (87.3%)	49 (98.0%)	0.041
Healing time (month)	3.69±1.01	2.37±1.06	0.000
Delayed union	19 (34.5%)	6 (12.0%)	0.011
Malunion	55 (100%)	0 (0%)	0.000
Self-reported Asymmetry	25 (45.5%)	0 (0%)	0.000
Numbness	0 (0%)	23 (46.0%)	0.000
VAS of pain	0.27±0.68	0.24±0.52	0.866
VAS of overall satisfaction	8.95±1.04	8.80±0.93	0.329
VAS of cosmetic satisfaction	7.93±1.93	8.06±1.46	0.935
Quick DASH score	12.49±3.04	12.12±2.48	0.671
Constant-Murley score	95.29±8.39	96.08±7.61	0.753
Returning to work	53 (96.4%)	49 (98.0%)	0.536
Time cost for returning to work (month)	3.34±1.37	1.47±0.89	0.000
Regaining ROM	41 (74.5%)	43 (86.0%)	0.324
Time cost for regaining ROM (month)	3.83±1.24	1.66±0.78	0.000
Regaining strength	31 (56.4%)	35 (70.0%)	0.107
Time cost for regaining strength (month)	8.03±2.78	3.86±2.45	0.000

### Fracture union

**Fig. 1:**
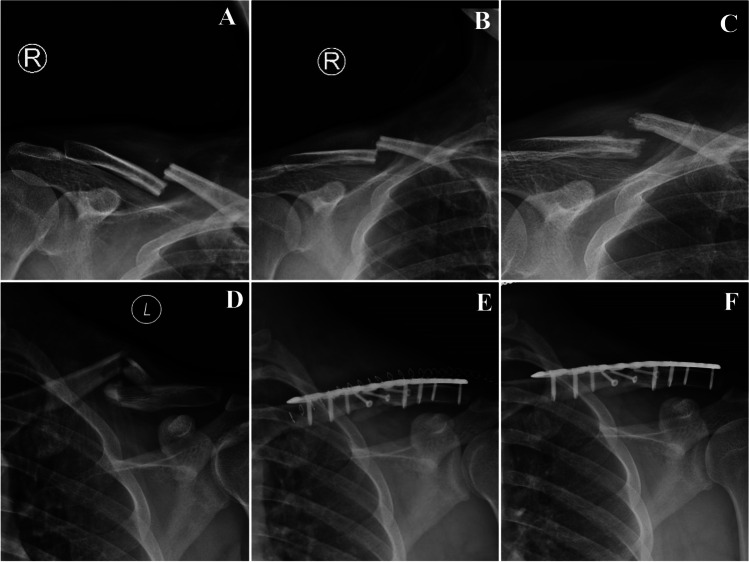
**A**. Anterior-posterior X-ray image immediately after injury of case 3 from conservative group. The facture was completely displaced. **B**. Anterior-posterior X-ray image after application of a “figure-of-8” brace and sling of case 3. The displacement was slightly corrected. **C**. Anterior-posterior X-ray image by the 10^th^ month after injury of case 3. The fracture was not united. **D**. Anterior-posterior X-ray image immediately after injury of case 4 from operative group. The facture was completely displaced. **E**. Anterior-posterior X-ray image of case 4 immediately after surgery. The facture achieved complete anatomic reduction. **F**. Anterior-posterior X-ray image by the 12^th^ months after injury of case 4. The facture achieved complete anatomic reduction but not united

**Fig. 2:**
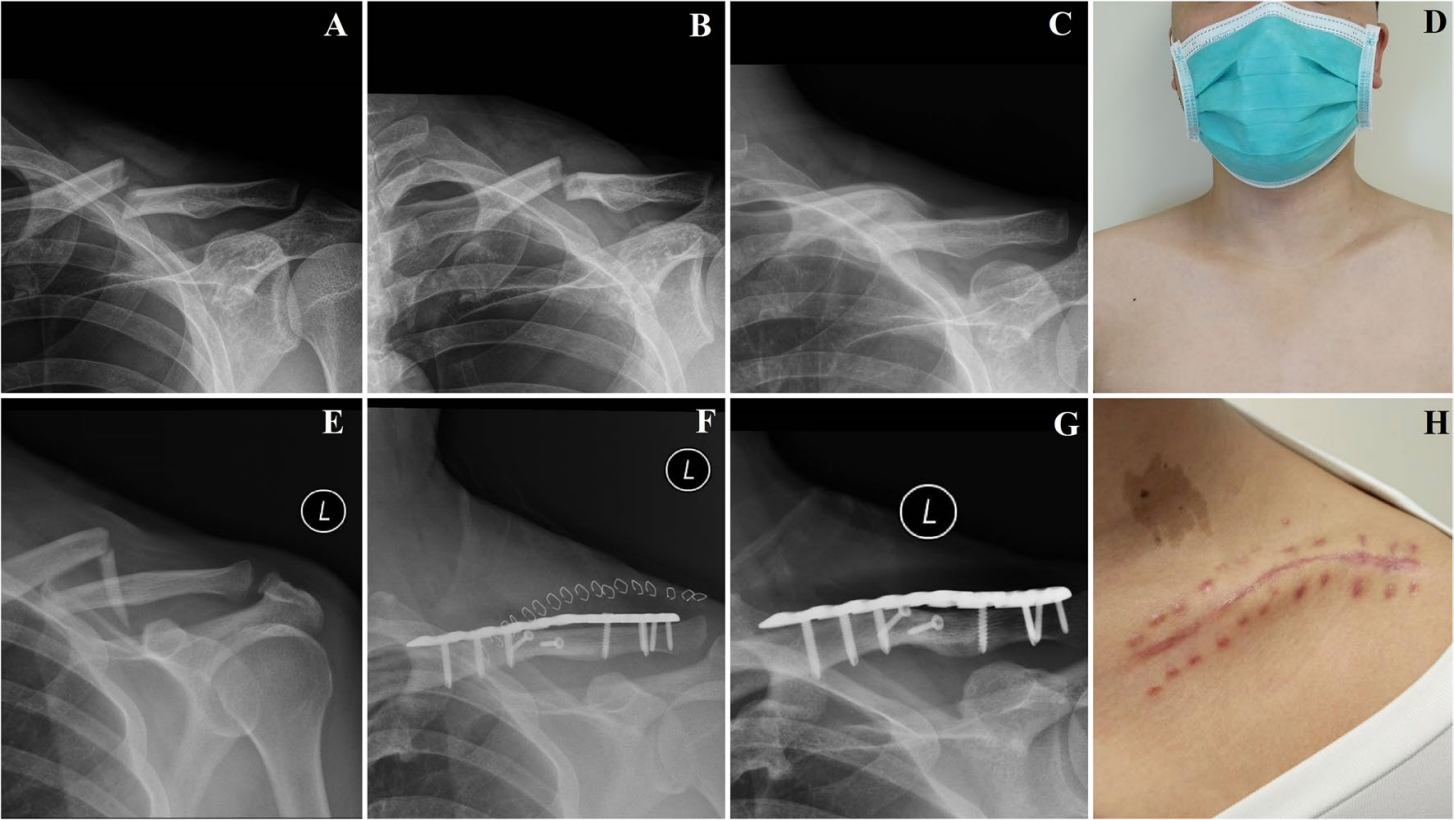
**A**. Anterior-posterior X-ray image immediately after injury of case 1 from conservative group. The facture was completely displaced. **B**. Anterior-posterior X-ray image after application of a “figure-of-8” brace and sling of case 1. The displacement was slightly corrected. **C**. Anterior-posterior X-ray image by the final follow-up of case 1. The facture achieved complete malunion. **D**. The gross photograph by the final follow-up of case 1. Asymmetry of shoulder and bump caused by malunion was obvious. **E**. Anterior-posterior X-ray image immediately after injury of case 2 from operative group. The facture was completely displaced. **F**. Anterior-posterior X-ray image of case 2 immediately after surgery. The facture achieved complete anatomic reduction. **G**. Anterior-posterior X-ray image by the final follow-up of case 2. The facture united with excellent alignment. **H**. The gross photograph by the final follow-up of case 2. No obvious malformation was detected but scar of incision was significant

In the operative group, the fracture healing rate was 98.0% (*n*=49), which was significantly higher than the non-operative group (87.3%, *n*=48, *P*=0.041). The non-operative group took longer to heal (3.69±1.01 months) compared to the operative group (2.37±1.06 months, *p*=0.000). Additionally, the delayed union rate in the non-operative group (34.5%) was significantly higher than the operative group (12.0%, *p*=0.011). The Patient with non-union in operative group (*n*=1, 2.0%) underwent additional surgery and eventually achieved bone healing. Rate of non-union in conservative group was higher (*n*=7, 12.7%), while none of those requested surgical fixation (Fig. 1). All patients in the non-operative group who achieved union reported radiographic malunion by the last follow-up, whereas this phenomenon was not observed in the operative group (Fig. 2). Moreover, all patients in the operative group who achieved union demonstrated satisfactory contraposition and alignment in x-ray image (*p*=0.000).

### Complications

Only one patient in the operative group reported a wound problem, which was attributed to a foreign body reaction to the suture, which was solved after removing suture. There were no reports of infection or other surgical side infection. The primary complication observed in the operative group was decrease of sensation around incision (*n*=23, 46%) during the follow-up period. In addition, no skin numbness was witnessed in the non-operative group (*p*=0.000). In operative group, most of patients (*n*=45, 90%) demanded implant removal within two years postoperatively, but only a few of these cases (*n*=5, 11.1%) reported hardware prominence, and the others made this decision merely due to worries about potential complications related to implant.

### Subjective score

By the final follow-up, the non-operative group had similar overall satisfactory scores (8.95±1.04) compared to the operative group (8.80±0.93, *p*=0.329). There was no significant difference in aesthetic score between the non-operative and operative groups (7.93±1.93 to 8.06±1.46, *p*=0.935). Similarly, there was no significant difference in pain score between the non-operative and operative groups (0.27±0.68 to 0.24±0.52, *p*=0.866).

### Objective score

Constant-Murley score and Quick-DASH score were similar between the non-operative and operative groups (95.29±8.39 to 96.08±7.61, *p*=0.753, and 12.12±2.48 to 12.12±2.48, *p*=0.671). There was no significant difference in the proportion of individuals returning to work (96.4% in the non-operative group and 98.0% in the operative group, *p*=0.536), the rate of regaining full range of motion (74.5% in the non-operative group and 86.0% in the operative group, *p*=0.111), and the probability of regaining strength (56.4% in the non-operative group and 70.0% in the operative group, *p*=0.107), although these figures were slightly higher in the operative group. The operative group required less time (3.34±1.37 months) to return to work compared to the non-operative group (1.47±0.89 months, *p*=0.000). The non-operative group took longer to regain full range of motion (3.83±1.24 months) compared to the operative group (1.66±0.78 months, *p*=0.000). Additionally, patients in the operative group regained strength more quickly (8.03±2.78 months) than patients in the non-operative group (3.86±2.45 months, *p*=0.000).

## Discussion

So far there has been little agreement about the management of replaced mid-shaft clavicle fracture. Both conservative and operative treatment has been recommended when reviewing previous studies. In this study, we have found that surgical fixation has higher union rate, shorter union time and takes less time to resume strength, movement and working ability when compared with conservative therapy. Also, operative treatment helps avoid malunion and asymmetry.

In the non-operative group, patients experienced a longer healing time, indicating that internal fixation might be helpful to maintain stability and fracture fragment contact and accelerate process of union, This consequence is consistent with the findings of Kumar (2022) [7], who reported a union rate of 60% within 12 weeks for operative patients, compared to only 28% for conservative patients.

In this study, we found that the rate of non-union in non-operative patients was significantly higher compared to operative patients, which aligns with previous research [9–12]. A displacement distance of more than 2cm has been considered a relative indication for surgery [13], but in our centre, conservative treatment was always an option regardless of the degree of displacement. Through strict immobilization in the early stages and a well-designed rehabilitation strategy, we were able to achieve a controlled non-union rate of 12.7%, which is lower than previously reported data [4]. It is worth noting that none of the non-operative patients with non-union opted for surgery, suggesting that the negative impact of this issue may be limited.

Given the nature of Robinson 2B type clavicle fractures, malunion is almost inevitable [14]. In our study, all non-operative patients who achieved bone healing showed signs of malunion. However, not all patients noticed this malformation, and less than half of the non-operative patients reported shoulder asymmetry. There is a limited amount of literature focusing on the cosmetic issues of clavicle fractures, but it is generally believed that cosmetic problems are mainly caused by incision wounds, particularly poor healing [15]. Incision scars can cause cosmetic problems and dissatisfaction among patients, but cosmetic problems of asymmetric shoulder caused by malunion has been ignored for a long time. In our research, we found no superior cosmetic satisfaction in conservative groups at the final follow-up. It is important to note that less than half of non-operative patients noticed shoulder asymmetry, while all operative patients had incision scars. This suggests that the cosmetic problem caused by malunion may have been underestimated.

The incidence of complications varies among different studies mainly due to the vague definition of “complication”, and the conclusion could differ among studies [16]. In our study, complications of conservative and operative group were discussed separately, and common complications were investigated further to reveal possible mechanism. For a long time, complications of surgery have been primarily associated with grafts and incisions, with skin numbness being rarely reported [17]. Skin numbness occurs as a result of damage to the supra-clavicle and cutaneous nerves during the operation, which can occur around the surgical site or in other areas of the shoulder, but it can theoretically be caused by the bump pressure of malunion as well [18]. Despite efforts to protect the nerves, it is challenging to keep them intact due to anatomical variations and the need for surgical field exposure. In our study, nearly half of operative patients had decreased sensation of shoulder skin, which is not seen in non-operative group, suggesting that this complication is mainly the consequence of iatrogenic injury instead of mechanical pressure. Among those cases with numbness, some recovered after one year, while others persisted. We have not yet found convinced evidence of its negative effect on satisfactory, and this field leaves much for further study.

At the finial follow-up, patients in both groups had similar function scores, including Quick-DASH and Constant-Murley. The former scale focus on the performance in daily life, and another none mainly reflects motion of shoulder. Scores in both groups were quite high, indicating that both conservative and operative treatment for mid-shaft clavicle fracture could achieve a satisfactory consequence. This conclusion is consistent with some previous literature [19–21]. However, some other studies have declared that surgical treatment brings better outcomes than conservative treatment [17, 22]. We would like to attribute this difference to different length of follow-up in various researches, as the advantage of surgery is typically reflected in the early stages [23]. In our study, as the follow-up time is relatively longer than most of previous articles, we suppose that the advantage in clinical function of operative might be eliminated by time.

This study also examined three main areas that reflect the impact on daily life and the operative group showed significant faster recovery. Nowadays, intelligent office technology has reduced reliance on the shoulder, allowing most patients with mildly limited shoulder motion to be competent for work tasks, but this seemed still difficult for conservative patients. The shorter gap between injury and returning to work give the patients competitive edge in this highly efficient society. Our study showed that the main challenge in regaining ROM was supination, and the reasons differed between the two groups. Non-operative patients experienced shoulder constraint, likely due to the shortening of the affected clavicle, while operative patients could not identify the reason and often attributed it to the plate, even though the hardware was not prominent. According to our investigation, patients faced difficulties in strength training were primarily concerned about the risk of a second fracture (non-operative patients) or plate breakage (operative patients). We suppose that difference in time required for recovery was due to the longer immobilization and severe pain in early stage for non-operative patients and shoulder stiffness thus caused.

## Conclusion

In conclusion, for complete displaced and comminute clavicle fracture, surgery provides with faster and more reliable union, and shorter time in recovery.

## Supplementary information

ESM 1 (SAV 7 kb)

## Data Availability

The medical records and original image used during this study are available from the corresponding author on reasonable request.
